# A 16-year Longitudinal Cohort Study of Incidence and Bacteriology of Necrotising Fasciitis in England

**DOI:** 10.1007/s00268-020-05559-2

**Published:** 2020-05-07

**Authors:** David M. S. Bodansky, Irena Begaj, Felicity Evison, Mark Webber, Ciaran B. Woodman, Olga N. Tucker

**Affiliations:** 1 The Departments of Surgery, Royal Liverpool and Broadgreen University Hospitals Trust, Prescot Street, Liverpool, L7 8XP UK; 2 Health Informatics and Surgery, University Hospitals Birmingham NHS Foundation Trust, Birmingham, B15 2GW UK; 3 Quadram Institute Bioscience, Norwich, NR4 7UQ UK; 4 Cancer Sciences, University of Birmingham, Birmingham, B15 2SG UK

## Abstract

**Background:**

Necrotising fasciitis (NF) is a rapidly progressive, destructive soft tissue infection with high mortality. The primary aim of this study was to evaluate the incidence and mortality of NF amongst patients admitted to English National Health Service (NHS) hospitals. The secondary aims included the identification of risk factors for mortality and causative pathogens.

**Methods:**

The Hospital Episodes Statistics database identified patients with NF admitted to English NHS Trusts from 1/1/2002 to 31/12/2017. Information on patient demographics, co-morbid conditions, microbiology specimens, surgical intervention and in-hospital mortality was collected. Uni- and multivariable analyses were performed to investigate factors related to in-hospital mortality.

**Results:**

A total of 11,042 patients were diagnosed with NF. Age-standardised incidence rose from 9 per million in 2002 to 21 per million in 2017 (annual percentage change = 6.9%). Incidence increased with age and was higher in men. Age-standardised mortality rate remained at 16% over the study period, while in-hospital mortality declined. On multivariable analysis, the following factors were associated with increased risk of in-hospital mortality: emergency admission, female sex, history of congestive heart failure, peripheral vascular disease, chronic kidney disease and cancer. Admission year and diabetes, which was significantly prevalent at 27%, were not associated with increased risk of mortality. Gram-positive pathogens, particularly Staphylococci, decreased over the study period with a corresponding increase in Gram-negative pathogens, predominantly *E. coli*.

**Conclusion:**

The incidence of NF increased markedly from 2002 to 2017 although in-hospital mortality did not change. There was a gradual shift in the causative organisms from Gram-positive to Gram-negative.

## Introduction

Necrotising fasciitis (NF) is a rapidly progressing soft tissue infection that frequently results in permanent disability and death despite parenteral antibiotic therapy and aggressive surgical management [1–4]. NF usually starts as a local infection from an abrasion, scratch or bite [5, 6]. The patient may present with cellulitis, which rapidly progresses with pain disproportionate to the area of infection [7]. The infection spreads to the fascial layer and then laterally along this plane with superficial tissue necrosis. Diabetes, renal disease and increasing age have been associated with poor outcomes [8, 9].

Necrotising fasciitis may be caused by a variety of aerobic and facultative anaerobic bacterial species, but is frequently polymicrobial. As many as 4 or 5 species may be cultured and the contribution of each to the pathogenesis of the disease is often not clear [1, 10]. Infections have been divided into three categories determined by the isolated species into polymicrobial, Group A streptococcal (80–90%) and Gram-negative rods infections [8, 11–13].

The incidence of NF has been described in the USA, with 4.8 deaths per 1,000,000 person years without a change in incidence between 2002 and 2013 [8]. In this study, by Arif and colleagues of 9871 cases of NF, streptococcal species were identified in 48% (260/546) deaths with a microbiological diagnosis, staphylococcal species in 22% (119/546) and Gram-negative species in 21% (114/546). In this study, diabetes, renal disease and obesity were associated with mortality. An increase in incidence has been reported in New Zealand from 0.18 per 100,000 person-years in 1990 to 1.69 in 2006 and mortality from 0 to 0.3 per 100,000 person-years. In this latter study, disease risk was the highest in the elderly, males, and Pacific and Maori populations; however, microbiology was not reported [14]. There are no studies describing the incidence in England, where the estimated incidence and microbiology are unknown [1, 14–18].

The primary aim of this study was to determine the incidence and associated mortality of NF in England. The secondary aims were to investigate risk factors for mortality and causative pathogens in patients admitted with a diagnosis of NF in English NHS Hospitals over the study period.

## Methods

The Clinical Audit Committee of the University Hospitals Birmingham NHS Foundation Trust approved the study. Patients were identified from the Hospital Episode Statistics (HES) database, with those admitted to an English NHS Hospital, who were discharged between 1 January 2002 and 31 December 2017. Patients with a diagnosis of NF were identified using the tenth revision of the International Classification of Disease (ICD-10) code M72.5 assigned to the diagnostic fields of the first episode of each admission and M72.6 when introduced in 2008 [19]. Transfers between hospitals (434 patients) and readmissions with the same diagnosis were excluded. Pre-specified patient level data items for collection were identified and extracted from HES to include the date of admission and discharge, the hospital attended, demographic characteristics (age, gender, ethnicity, socio-economic status by postcode), diagnostic (co-morbid conditions) and procedural (surgical debridement, fasciotomy, skin grafting) codes, microbiological samples taken (using ICD-10 codes) and in-hospital mortality. Search terms and synonyms for procedural interventions were selected from the Office of Population, Censuses and Surveys Classification of Surgical Operations and Procedures, 4th revision (OPCS-4) [20] (listed in Appendix A). The subgroup of patients admitted as an emergency who underwent surgery for NF were analysed.

## Statistical methods

Age-standardised incidence rates were calculated using the European Standard Population 2013. Change over time was monitored using annual population change statistics obtained from ONS mid-year population estimates following the 2011 census. Data on diabetes mellitus (DM) prevalence were obtained through the Quality and Outcome Framework (QOF) [21]. Incidence rates of NF in the population with DM were determined using HES and QOF data. Social deprivation was classified by postal code (English Indices of Deprivation, Ministry of Housing, Communities and Local Government). In-hospital mortality was measured. Patients with missing gender and age were removed from the data set. Unknown categories for ethnicity and deprivation were included. Patients with missing microbiological data were excluded in the analysis of changes in the proportions of causative organisms. Univariable analysis was performed to identify factors influencing in-hospital mortality to include in a multivariable model. All analyses were conducted using Stata SE v13 [22].

## Internal validation

To confirm the accuracy of NF diagnoses, a random sample of 12 hospitals was chosen from the 160 acute hospitals in England to provide a representative sample of the complete cohort. Patients with a diagnosis of NF (M72.5 in ICD-10) who underwent in-hospital surgical debridement were randomly selected at each of the 12 hospitals over the 10-year study period using a computer-generated method by a trained, blinded coder at each Trust’s coding department. A blinded Consultant Microbiologist at each hospital reviewed the microbiology and pathology records for tissue samples taken at surgery for each patient to determine the accuracy of the diagnosis. Pre-specified patient-level data were collected using an anonymised questionnaire. A diagnosis of NF was accepted as accurate in the presence of a positive culture of one or more pathogens known to cause NF from local tissue swabs and/or blood cultures, or histological necrosis in debrided fascial and subcutaneous local tissue at surgery or autopsy.

## Results

### Data accuracy and missing data

Initially, 14,659 admissions to hospital for NF were identified between 1 January 2002 and 31 December 2017, using the ICD10 code M72.5 and M72.6. Missing patient age or gender excluded 16 admissions. A further 285 admissions were excluded because they were not resident in England, and thus follow-up data were not reliable. There were 2067 patients who had multiple admissions, so only the first was kept for analysis, excluding 3314 admissions, leaving 11,042 patients. Coding completion for ethnicity improved over the study period from 72.6% in 2002 to 94% in 2017.

The validation study provided 275 randomly selected patients with an ICD-10 diagnosis of NF, which represented 4% of the total cohort who underwent in-hospital surgical debridement. In total, 212 patients had a record of a wound swab or tissue sampling at surgery of which 179 (81%) had microbiological and/or pathological confirmation of NF.

### Patient characteristics

There were 11,042 patients in the study with a median age at diagnosis of 58 years (IQR 26). There were 5819 (52.7%) males, and the median male age was 57 (IQR 25) compared with 59 for females (IQR 28) (Table 1). The ethnic structure reflected that of the UK population (Table 1) [23]. There were 3111 patients (28.2%) from the most deprived socio-economic quintile, compared with 1610 (14.6%) from the least deprived (Table 1).

**Table 1:** Patient demographics

		Patients (%)
Gender	Male	5819 (52.7)
	Female	5223 (47.3)
Age group	Under 10	195 (1.8)
	10–19	152 (1.4)
	20–29	532 (4.8)
	30–39	1170 (10.6)
	40–49	1773 (16.1)
	50–59	2070 (18.7)
	60–69	2223 (20.1)
	70–79	1743 (15.8)
	80 +	1184 (10.7)
Ethnicity	White	9771 (88.5)
	Asian	368 (3.3)
	Black	279 (2.5)
	Chinese, Other	186 (1.7)
	Unknown	438 (4.0)
Deprivation quintile	1—Most deprived	3111 (28.2)
	2	2413 (21.9)
	3	1971 (17.9)
	4	1832 (16.6)
	5—Least deprived	1610 (14.6)
	Unknown	105 (1.0)
	Diabetes	2941 (26.6)
	Peripheral vascular disease	1537 (13.9)
	Pulmonary disease	1514 (13.7)
Co-morbidities	Chronic kidney disease	1134 (10.3)
	Congestive heart failure	809 (7.3)
	Cancer	1134 (10.3)
	Cellulitis with previous 30 days	640 (5.8)
Location	Ankle and foot	533 (4.8)
	Arm—Unspecified	59 (0.5)
	Forearm	279 (2.5)
	Hand	218 (2.0)
	Leg	240 (2.2)
	Lower leg	1271 (11.5)
	Multiple sites	868 (7.9)
	Other—including trunk, head etc.^a^	2855 (25.9)
	Pelvic region and thigh	3029 (27.4)
	Shoulder region	120 (1.1)
	Upper arm	178 (1.6)
	Unspecified	1392 (12.6)

^a^It is not possible to break down this division further

The most common co-morbidities were diabetes (2941; 26.6%), peripheral vascular disease (PVD) (1537; 13.9%) and pulmonary disease (1514; 13.7%) (Table 1). The pelvic region and thigh were the most common site for NF (3029; 27.4%) followed by the lower leg (1271; 11.5%) (Table 1). Only 640 patients (5.8%) had been admitted with cellulitis in the 30 days preceding their NF admission. In total, 1551 (14.0%) patients had been treated for cellulitis previously in hospital.

### Incidence and mortality of necrotising fasciitis

**Fig. 1:**
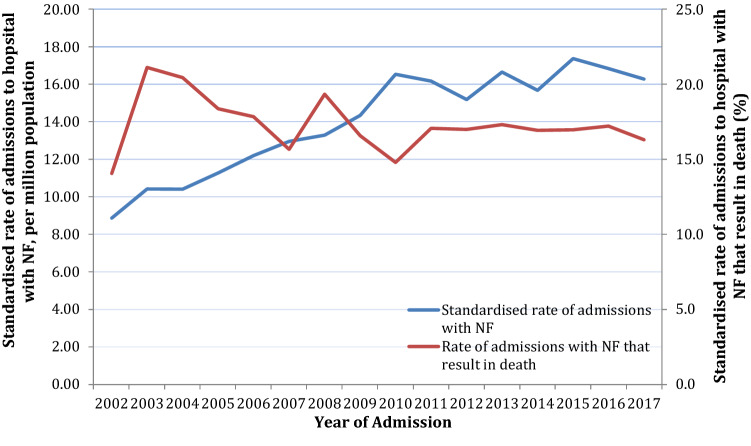
Age standardised rate of admissions to hospital for patients with necrotising fasciitis and of mortality. The rate of admissions rose from 9 per million population in 2002 to 16 per million in 2017. The rate of admissions resulting in death remained constant across the study period

The age-standardised rate of admissions of NF doubled from 2002 to 2017 (*p* < 0.001, Fig. 1). This increase was observed in both sexes and was higher for men at all ages than women, with rate of admission increasing with age. The rate of mortality of patients admitted with NF across the study period was 16% and did not change statistically (*p* = 0.237).

A multivariable analysis showed that in-hospital mortality was higher for women (OR 1.28; 1.16–1.41, *p* < 0.0001) yet was not significantly higher at 1 year (*p* = 0.573) compared to men. Patients from the most deprived socio-economic quintile had the highest rate of 1-year mortality. Co-morbidities significantly increased in-hospital mortality with the exception of diabetes, which did not increase the in-hospital mortality (OR 1.01; 0.91–1.11, *p* = 0.9042) (Table 2). Although most patients were emergency admissions, there were 951 elective hospital patients who later developed NF. Necrotising fasciitis of the extremities (the feet or hands) were associated with increased survival by one year (Table 3).

**Table 2:** Demographic and co-morbidity Multivariable analysis

		In hospital mortality		Deaths within 30 days		Deaths within 90 days		Deaths within 1 year	
Gender	Male	1.00 (reference)		1.00 (reference)		1.00 (reference)		1.00 (reference)	
	Female	1.28 (1.16, 1.41)	<0.0001	1.05 (0.96, 1.15)	0.31	1.00 (0.91, 1.09)	0.949	0.97 (0.89, 1.07)	0.573
Age group	Under 10	0.07 (0.03, 0.16)	<0.0001	0.08 (0.04, 0.15)	<0.0001	0.07 (0.04, 0.13)	<0.0001	0.06 (0.03, 0.10)	<0.0001
	10–19	0.09 (0.04, 0.22)	<0.0001	0.08 (0.04, 0.16)	<0.0001	0.05 (0.02, 0.11)	<0.0001	0.04 (0.02, 0.09)	<0.0001
	20–29	0.16 (0.11, 0.23)	<0.0001	0.14 (0.10, 0.19)	<0.0001	0.11 (0.08, 0.15)	<0.0001	0.10 (0.07, 0.13)	<0.0001
	30–39	0.31 (0.25, 0.39)	<0.0001	0.27 (0.22, 0.33)	<0.0001	0.22 (0.19, 0.27)	<0.0001	0.21 (0.18, 0.26)	<0.0001
	40–49	0.42 (0.35, 0.50)	<0.0001	0.39 (0.33, 0.46)	<0.0001	0.35 (0.30, 0.40)	<0.0001	0.31 (0.27, 0.37)	<0.0001
	50–59	0.67 (0.58, 0.78)	<0.0001	0.66 (0.58, 0.76)	<0.0001	0.61 (0.53, 0.70)	<0.0001	0.55 (0.48, 0.63)	<0.0001
	60–69	1.00 (reference)		1.00 (reference)		1.00 (reference)		1.00 (reference)	
	70–79	1.40 (1.21, 1.61)	<0.0001	1.46 (1.27, 1.67)	<0.0001	1.67 (1.45, 1.92)	<0.0001	1.74 (1.51, 2.01)	<0.0001
	80 +	2.66 (2.27, 3.11)	<0.0001	3.78 (3.20, 4.46)	<0.0001	4.69 (3.91, 5.63)	<0.0001	4.79 (3.96, 5.79)	<0.0001
Ethnicity	White	1.00 (reference)		1.00 (reference)		1.00 (reference)		1.00 (reference)	
	Asian	0.89 (0.66, 1.20)	0.43	0.76 (0.57, 1.01)	0.058	0.67 (0.51, 0.89)	0.005	0.64 (0.49, 0.85)	0.002
	Black	0.68 (0.47, 0.98)	0.037	0.55 (0.39, 0.77)	0.001	0.59 (0.43, 0.81)	0.001	0.64 (0.47, 0.87)	0.005
	Chinese, Other	0.78 (0.50, 1.20)	0.257	0.88 (0.60, 1.31)	0.538	0.87 (0.60, 1.27)	0.478	0.80 (0.55, 1.18)	0.264
	Unknown	3.81 (3.06, 4.74)	<0.0001	2.66 (2.12, 3.33)	<0.0001	1.97 (1.56, 2.48)	<0.0001	1.63 (1.29, 2.07)	<0.0001
Deprivation quintile	1—Most deprived					1.00 (reference)		1.00 (reference)	
	2					0.93 (0.81, 1.05)	0.244	0.91 (0.79, 1.03)	0.139
	3					0.92 (0.80, 1.06)	0.238	0.88 (0.77, 1.01)	0.075
	4					0.77 (0.67, 0.89)	<0.0001	0.76 (0.66, 0.88)	<0.0001
	5—Least deprived					0.76 (0.66, 0.89)	<0.0001	0.75 (0.65, 0.87)	<0.0001
	Unknown					0.70 (0.46, 1.06)	0.094	0.68 (0.45, 1.02)	0.065
	Diabetes	0.87 (0.78, 0.97)	0.014					1.29 (1.16, 1.44)	<0.0001
	Peripheral vascular disease			1.23 (1.08, 1.41)	0.001	1.23 (1.08, 1.39)	0.002	1.19 (1.05, 1.36)	0.008
	Pulmonary disease			1.13 (0.99, 1.29)	0.07	1.17 (1.02, 1.33)	0.023	1.22 (1.07, 1.40)	0.003
Co-morbidities	Chronic kidney disease	2.47 (2.14, 2.85)	<0.0001	2.59 (2.24, 3.01)	<0.0001	2.74 (2.34, 3.20)	<0.0001	2.53 (2.16, 2.98)	<0.0001
	Congestive heart failure	3.23 (2.74, 3.82)	<0.0001	2.79 (2.34, 3.33)	<0.0001	2.88 (2.38, 3.49)	<0.0001	3.10 (2.54, 3.78)	<0.0001
	Cancer	2.08 (1.81, 2.40)	<0.0001	5.86 (5.02, 6.84)	<0.0001	6.35 (5.36, 7.52)	<0.0001	6.52 (5.46, 7.78)	<0.0001
	Cellulitis with previous 30 days			1.52 (1.28, 1.79)	<0.0001	1.74 (1.48, 2.06)	<0.0001	1.71 (1.44, 2.02)	<0.0001
	Previous Cellulitis	0.82 (0.66, 1.02)	0.073	0.69 (0.54, 0.89)	0.004	0.58 (0.45, 0.75)	<0.0001	0.62 (0.48, 0.79)	<0.0001

**Table 3:** Location and admission year multivariable analysis

		In hospital mortality		Deaths within 30 days		Deaths within 90 days		Deaths within 1 year	
Location of NF	Ankle and foot	0.25 (0.18, 0.35)	<0.0001	0.36 (0.28, 0.47)	<0.0001	0.41 (0.32, 0.52)	<0.0001	0.37 (0.29, 0.47)	<0.0001
	Leg	1.30 (0.93, 1.83)	0.125	1.10 (0.79, 1.52)	0.578	1.05 (0.76, 1.43)	0.783	1.14 (0.84, 1.56)	0.403
	Lower leg	1.14 (0.96, 1.34)	0.13	1.01 (0.86, 1.19)	0.881	0.97 (0.83, 1.14)	0.692	0.91 (0.77, 1.07)	0.234
	Pelvic region and thigh	1.00 (reference)		1.00 (reference)		1.00 (reference)		1.00 (reference)	
	Arm including hand	0.36 (0.23, 0.54)	<0.0001	0.36 (0.25, 0.52)	<0.0001	0.37 (0.26, 0.52)	<0.0001	0.41 (0.30, 0.57)	<0.0001
	Forearm	0.74 (0.53, 1.04)	0.079	0.82 (0.60, 1.11)	0.195	0.77 (0.57, 1.04)	0.092	0.67 (0.49, 0.92)	0.012
	Upper arm	1.37 (0.93, 2.01)	0.11	0.96 (0.66, 1.41)	0.846	0.93 (0.64, 1.35)	0.714	0.82 (0.56, 1.19)	0.293
	Shoulder region	1.28 (0.79, 2.05)	0.313	0.78 (0.48, 1.26)	0.305	0.72 (0.45, 1.13)	0.156	0.67 (0.43, 1.06)	0.086
	Multiple sites	2.33 (1.95, 2.79)	<0.0001	1.84 (1.54, 2.20)	<0.0001	1.61 (1.34, 1.92)	<0.0001	1.49 (1.24, 1.79)	<0.0001
	Other—incl trunk, head and neck	0.84 (0.73, 0.96)	0.01	0.81 (0.71, 0.92)	0.001	0.75 (0.67, 0.85)	<0.0001	0.73 (0.64, 0.82)	<0.0001
	Unspecified	1.51 (1.29, 1.77)	<0.0001	1.31 (1.13, 1.53)	0.001	1.15 (0.98, 1.34)	0.084	1.09 (0.93, 1.27)	0.302
Year of Admission	2002	1.00 (reference)		1.00 (reference)		1.00 (reference)		1.00 (reference)	
	2003	1.22 (0.88, 1.70)	0.233	0.98 (0.71, 1.34)	0.878	1.14 (0.83, 1.57)	0.433	0.99 (0.72, 1.37)	0.953
	2004	1.00 (0.71, 1.39)	0.982	0.87 (0.64, 1.20)	0.399	0.88 (0.64, 1.21)	0.433	0.80 (0.58, 1.11)	0.184
	2005	1.19 (0.86, 1.65)	0.295	1.19 (0.87, 1.63)	0.271	1.08 (0.79, 1.48)	0.641	0.97 (0.70, 1.34)	0.861
	2006	1.00 (0.72, 1.38)	0.994	0.85 (0.62, 1.16)	0.302	0.89 (0.65, 1.21)	0.462	0.79 (0.57, 1.08)	0.135
	2007	0.94 (0.68, 1.30)	0.718	0.81 (0.59, 1.09)	0.165	0.78 (0.58, 1.06)	0.115	0.59 (0.43, 0.80)	0.001
	2008	1.15 (0.84, 1.57)	0.392	0.86 (0.63, 1.16)	0.313	0.90 (0.67, 1.22)	0.511	0.62 (0.45, 0.84)	0.002
	2009	0.93 (0.68, 1.27)	0.64	0.67 (0.50, 0.91)	0.01	0.73 (0.54, 0.98)	0.038	0.45 (0.33, 0.61)	<0.0001
	2010	0.79 (0.58, 1.08)	0.136	0.64 (0.48, 0.86)	0.003	0.62 (0.46, 0.83)	0.001	0.38 (0.28, 0.51)	<0.0001
	2011	0.89 (0.65, 1.21)	0.46	0.69 (0.52, 0.93)	0.014	0.74 (0.55, 0.99)	0.042	0.40 (0.29, 0.54)	<0.0001
	2012	0.90 (0.66, 1.23)	0.509	0.75 (0.56, 1.01)	0.058	0.71 (0.53, 0.95)	0.023	0.38 (0.28, 0.51)	<0.0001
	2013	0.91 (0.67, 1.23)	0.546	0.71 (0.54, 0.95)	0.023	0.54 (0.41, 0.73)	<0.0001	0.29 (0.21, 0.39)	<0.0001
	2014	0.82 (0.60, 1.12)	0.208	0.65 (0.49, 0.87)	0.004	0.46 (0.34, 0.62)	<0.0001	0.24 (0.18, 0.33)	<0.0001
	2015	0.90 (0.67, 1.22)	0.513	0.77 (0.58, 1.02)	0.072	0.47 (0.35, 0.63)	<0.0001	0.25 (0.19, 0.34)	<0.0001
	2016	0.84 (0.62, 1.14)	0.272	0.60 (0.45, 0.80)	0.001	0.33 (0.25, 0.45)	<0.0001	0.17 (0.13, 0.24)	<0.0001
	2017	0.93 (0.69, 1.27)	0.663	0.52 (0.39, 0.69)	<0.0001	0.29 (0.21, 0.39)	<0.0001	0.15 (0.11, 0.20)	<0.0001

### Emergency admissions requiring surgery

Of the subgroup of emergency admissions receiving surgery, there were 6764 patients in total with a median age of 57 (IQR 44–68 years). There were 3723 men (55.0%), and the median male age was 56 (IQR 44–68) compared to 57 years for women (IQR 44–69). The ethnic structure was similar to the UK population with similar numbers of co-morbidities (Table 1).

Surgical intervention became more common over the study period with 190 patients receiving an intervention (debridement, amputation or grafting) in 2002 compared to 602 in 2017. However, the timing of surgical intervention from admission remained constant across the study period with the median time from admission to surgery being 1 day: debridement 1 day; to amputation 1 day and skin grafting 12 days (IQR 5–22).

Patients who underwent surgery on the same day as admission were more likely to die during their admission than those operated on later (23.1% vs. 20.2%, *p* = 0.006) after accounting for all other factors (co-morbidities, age, socio-economic deprivation, etc.). This remained true for deaths at 30 and 90 days.

### Incidence and mortality for surgical patients

**Fig. 2:**
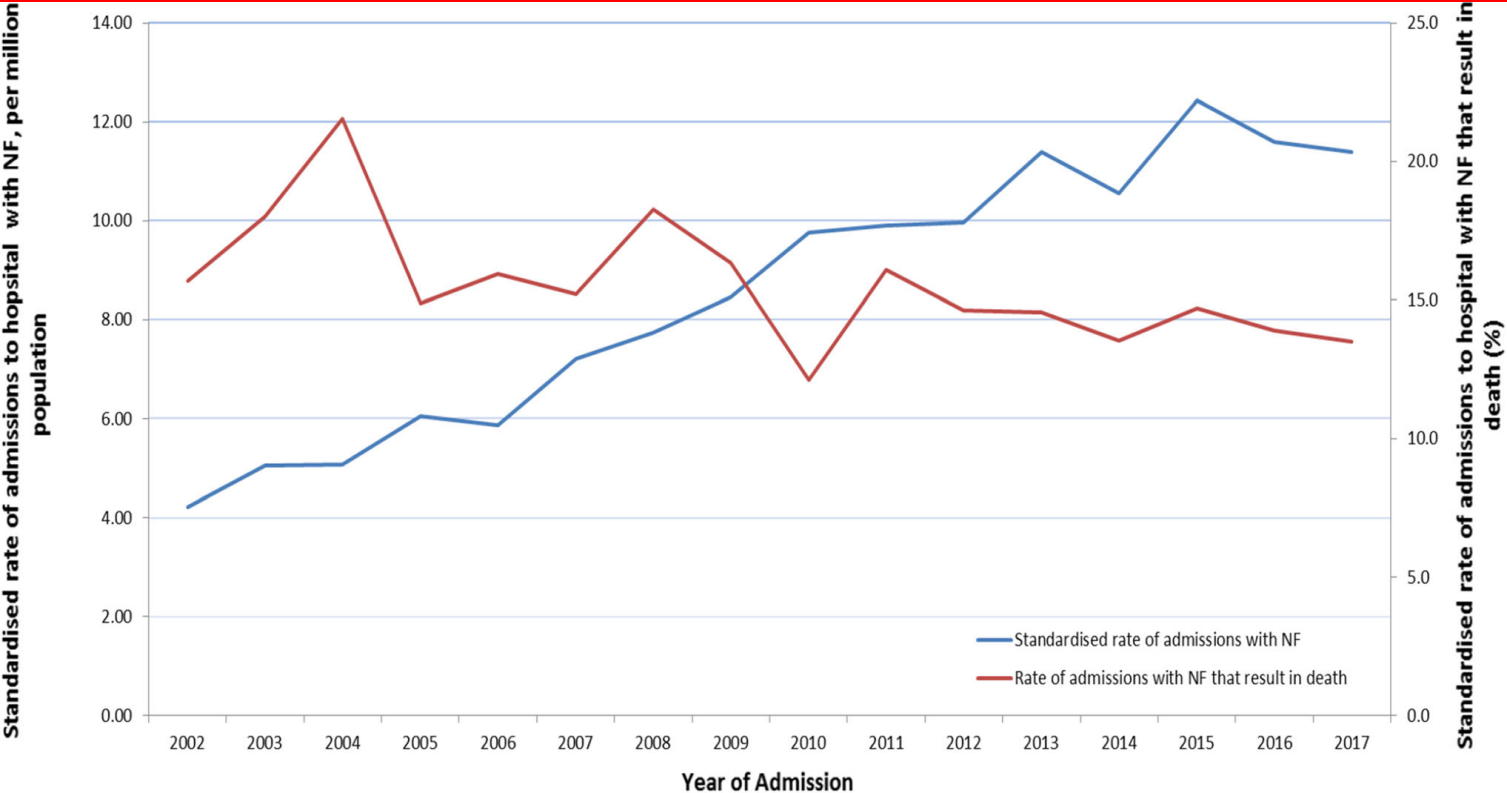
Age standardised rate of admissions and of mortality for emergency admissions of patients with necrotising fasciitis requiring surgery. For those patients with necrotising fasciitis receiving surgery, the rate of admission rose from 4 per million population in 2002 to 12 per million population in 2017. However the standardised rate of admissions that resulted in death fell during the study period

Age-standardised incidence for NF patients requiring surgery increased from 4 to 20 per million across the study period (Fig. 2). However, the mortality rate of NF remained constant across the study period (*p* = 0.19). Age-specific rate of admission to hospital with NF was higher for men than women at all ages.

### Diabetes

GP population data on diabetes were available from 2007 to 2017. During this time, the GP diabetic population rose from 3.7 to 6.7%. However, the admissions of NF with diabetes rose from 25.4% in 2007 to 34.5% in 2017.

### Microbiological characteristics

**Fig. 3:**
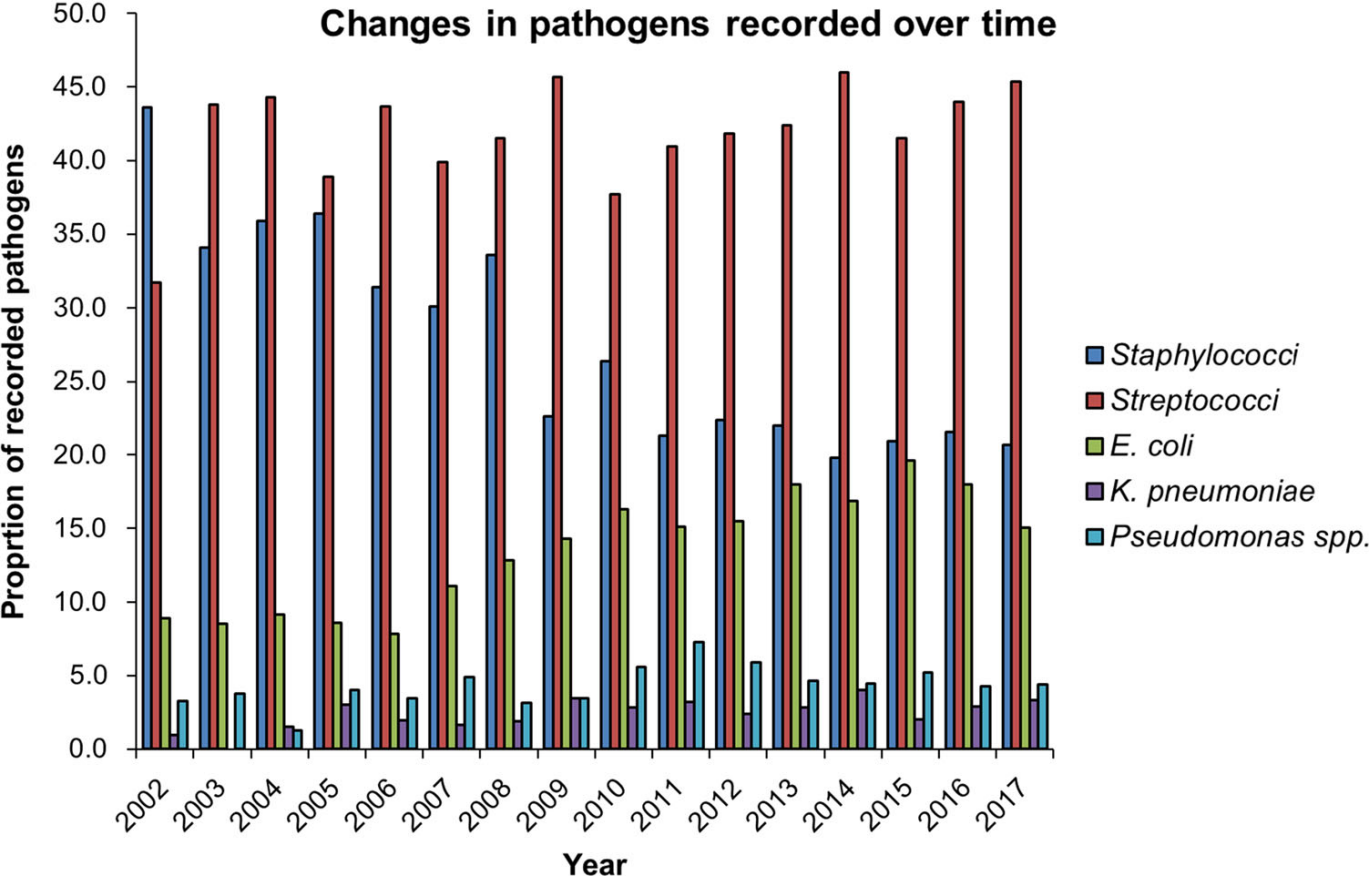
Change in pathogens from patients receiving surgery over time. Although the proportion of Gram-positive organisms overall fell during the study period, streptococci specii remained high whilst staphylococci specii dropped. Gram-negative isolated specii became more common, in particular *E. coli*

An identified pathogen was recorded in 4446 (40.3%) patients. Of surgical patients, 3306 patients (48.9)% had an identified pathogen compared to 1140 (26.6%) patients treated non-surgically. Gram-positive species represented 63.71% of all isolated pathogens (range per year from 55.9 to 75.7%) (Fig. 3). There was a reduction in the overall proportion of Gram-positive species isolated over time from 74.1% of all isolated pathogens in 2002 to 65.3% in 2017. This was largely due to a decrease in isolation of staphylococci, where from intra-operative samples numbers fell 44.3% of all recorded pathogens in 2002 to 21.1% in 2017 (Fig. 3). An increase in the overall proportion of Gram-negative species isolated from intra-operative samples rose from 22.7% of all recorded pathogens in 2002 to 32.9% in 2017 due mainly to an increase in *E. coli* and *Klebsiella pneumoniae* species (Fig. 3). Isolation of anaerobes was low throughout the study period at 0.95% (range 0.3–1.7%) of all isolated species. No geographical hot spots of specific pathogens were identified.

Patients with Gram-negative species isolated had a mortality rate of 22.7% within hospital, compared to patients with Gram-positive species (*p* = 0.016). Patients with Gram-negative bacteriology also had higher mortality at 30 and 90 days and at 1 year than patients with Gram-positive species. In-hospital mortality was 22.7% (487) for patients with Gram-positive species, compared with 19.8% (444) for Gram-negative (*p* = 0.016). The 30-day mortality for Gram-positive species was 34.6% (741), but Gram-negative was 26.9% (604) (*p* < 0.0001). However, the 90-day mortality rose to 41.5% (890) for Gram-positive compared to 33.3% (749) for Gram-negative and by one year, the Gram-positive mortality rate was 44.0% (943), but Gram-negative was 36.3% (816) (*p* < 0.0001).

## Discussion

This study has demonstrated a marked increase in the incidence of NF admissions in England over a 16-year period from 2002 to 2017 (Fig. 1). The dates for the study period were chosen to evaluate 16 years of data and to encompass the census of 2011. In line with these findings, increased incidence of NF has been seen in other countries including New Zealand and USA [14]. The relationship to predisposing factors including DM, PVD, CKD, socio-economic status, sex and age is consistent with previous studies [2, 24].

Patients with diabetes (DM) are four times over-represented in the cohort, importantly; however, their mortality was no greater than the rest of the population with NF. Patients with DM increased by 62.3% over the study period in line with increasing prevalence of DM worldwide, with the UK population of patients with diabetes increased by 72.9% across the study period [25]. The observed increased incidence of NF cannot therefore be explained by increased prevalence of DM. The proportion of chronic diseases predisposing to NF has been compared to that in the UK population using the QOF in General Practice which has been previously validated but may underestimate the population [26–29].

An increase in the age-standardised death rate from NF was seen in line with the observed increased incidence (Fig. 1). Mortality remained constant across the study period. However, the age-standardised mortality rate reduced by 60% for patients admitted to hospital. An increased risk of in-hospital mortality was seen with emergency admission and in the presence of congestive heart failure, PVD and CKD. Although there has been a rise in the number of surgical interventions for patients admitted as an emergency, the time from admission to theatre remained unchanged over the study period. The rise in the number of patients undergoing operative intervention over the study period is likely due to increased awareness of NF and recognition of the value of early surgical debridement.

Of the cohort, 11,042 had ‘NF’ documented within patient medical records with 6764 undergoing surgery. Patients who underwent surgery on the day of admission had a higher 30- and 90-day mortality than those who underwent surgery after 24 h. This cohort likely represents more unwell patients with a poorer prognosis requiring immediate life-saving intervention. The reasons for management of patients without surgical intervention were not possible to elucidate in this study. It may be that these patients were unfit or died before surgery. Alternatively, they have had a severe soft tissue infection and been incorrectly coded as NF. The validation study of the surgical cohort in 12 centres confirmed over 80% of cases was correctly coded, suggesting that patients undergoing surgery did have a true diagnosis of NF.

Importantly, a change in isolated pathogenic species over the 10-year period was observed. Although the majority of isolates were staphylococci and streptococci species as in other studies, a falling number of isolated *Staphylococcus aureus* strains and an increase in Gram-negative species, predominantly *E. coli* and *Klebsiella pneumonia*, in the absence of a change in anaerobic species have been demonstrated. These data may reflect the effectiveness of anti-MRSA interventions across UK hospitals, which coincides with the study period [30, 31]. However, this pattern was also seen in patients presenting as an emergency from the community. Polymicrobial NF is the predominant form, and as many as 4 or 5 species may be cultured from cases of NF. The contribution of each isolated organism to the pathogenesis of the disease is often not clear [1, 3, 10]. Although infection is frequently polymicrobial, an increase in monomicrobial NF has also been described in other sites [1, 3, 10]. From HES data, it was not possible to identify a relative change in the incidence of monomicrobial and polymicrobial NF over time, which may have influenced the relative proportions of species isolated and underestimate responsible organisms. We are only able to comment about the total numbers of pathogens isolated from the cases of NF. The UK Standards for Microbiology Investigations guidelines for processing of samples recommend incubation of samples for investigation of skin, superficial and non-surgical wound swabs in blood agar [32]. Wound swabs from chronic ulcers, traumatic wounds and samples from abscesses and deep-seated wound infections should be cultured in Neomycin fastidious anaerobe agar with metronidazole 5 μg disc [33]. We cannot comment on the culture media and conditions used during sample processing over the study period as these were completed by microbiology laboratories across all study sites. Although isolation of anaerobes was low throughout the study period, this may reflect variation in culture techniques between centres. The findings therefore may underestimate anaerobic species.

The increase in Gram-negative infections pathogens isolated from NF cases has also been recently been reported elsewhere [34–36]. Interestingly, Lee et al. observed more frequent septic shock and higher risk of mortality in patients with Gram-negative monomicrobial NF [37]. It is important to note that the findings from that study were seen in one hospital over a 9-year period with a high incidence of isolated Gram-negative bacilli, predominantly Vibrio species, in 76.1% of patients. This represents an unusual aetiology in the UK and is likely associated with the different geographical location of the Lee study. The impact of alterations in isolated pathogenic species and the effect of polymicrobial compared to monomicrobial infections on outcome have not been formally evaluated. A change in the balance of pathogens causing NF may have implications for empirical antimicrobial therapy with Gram-negative species presenting the greatest current AMR challenge in hospital medicine.

There are several limitations of this observational approach. The observed increased incidence may be due to greater recognition of NF with improved levels of diagnoses and coding. Whilst the proportion of patients correctly coded in HES cannot be determined, previous publications support the use of routinely collected HES data for research with improving accuracy rates [14, 38]. Further, we validated our findings by auditing a random sample of 12 hospitals, which confirmed the accuracy of 81% of NF diagnoses for patients receiving surgery. To improve data accuracy and completeness, patients with missing data including gender and age and patients with missing microbiological data were excluded in the analysis of changes in the proportions of causative organisms.

Data on obesity and smoking were not collected, as these were not routinely collected data items in HES over the study period. In the UK, seven of ten British people will be overweight or obese by 2020 with 40% being obese by 2030 [39, 40]. As there is a clear association between rising levels of obesity and increasing prevalence of DM, obesity may be a contributing factor to the observed increased incidence of NF, but this could not be determined by this study.

It is clear that a number of aspects of patient care cannot be disclosed by the existing data sources including time of onset and duration of symptoms, severity and prognosis at presentation and diagnosis, management in the community, death in patients who never had surgery due to co-morbid disease or NF-related multi-organ failure, reasons for non-surgical management or choice of antimicrobials, their timing and frequency of administration. These factors impact on outcome, and changes in practice over time including earlier diagnosis, earlier admission to hospital for intervention and advances in intensive care management may explain the observed reduced in-hospital mortality.

## Conclusion

Our results show an increasing incidence of NF in England which is a cause for concern, but a reduction in in-hospital mortality. The time to surgical intervention did not change over the study period. These data support the establishment of a national database to allow an ongoing audit of the observed increased incidence and changes in pathogenic species, which would inform future treatment strategies and improved patient outcome.

## Appendix A: Surgical codes from OPCS-4

**Table:**

Amputation	X07	Amputation of arm
	X08	Amputation of hand
	X09	Amputation of leg
	X10	Amputation of foot
	X11	Amputation of toe
	N261	Total amputation of penis
	N262	Partial amputation of penis
Debridement	S541	Debridement of burnt skin of head or neck
	S551	Debridement of burnt skin NEC
	S561	Debridement of skin of head or neck
	S571	Debridement of skin NEC
	S573	Toilet of skin NEC
	S581	Larvae debridement therapy of skin of head or neck
	S582	Larvae debridement therapy of skin NEC
	T774	Debridement of muscle NEC
	T963	Debridement of soft tissue NEC
	Y055	Debridement of organ NOC
Graft	S35	Split autograft of skin
	S36	Other autograft of skin
	S37	Other graft of skin
	S38	Graft of mucosa
	S39	Graft of other tissue to skin
Dressing	S544	Dressing of burnt skin of head or neck NEC
	S545	Attention to dressing of burnt skin of head or neck
	S547	Dressing of burnt skin of head or neck using vacuum-assisted closure device
	S554	Dressing of burnt skin NEC
	S555	Attention to dressing of burnt skin NEC
	S557	Dressing of burnt skin using vacuum-assisted closure device NEC
	S564	Dressing of skin of head or neck NEC
	S565	Attention to dressing of skin of head or neck NEC
	S567	Dressing of skin of head or neck using vacuum-assisted closure device
	S574	Dressing of skin NEC
	S575	Attention to dressing of skin NEC
	S577	Dressing of skin using vacuum-assisted closure device
Drainage	H58	Drainage through perineal region
	P14	Incision of introitus of vagina
	T34	Open drainage of peritoneum
	N244	Incision of male periurethral tissue
	P131	Drainage of female perineum
	X125	Drainage of amputation stump
	N323	Incision of penis (NEC)
	N322	Drainage of penis
	S471	Drainage of lesion of skin of head or neck
	S472	Drainage of lesion of skin NEC
	S473	Incision of lesion of skin of head or neck
	S474	Incision of lesion of skin NEC
	S475	Incision of skin of head or neck
	S476	Incision of skin NEC
Fasciotomy	T55	Release of fascia
